# Costs and Benefits of Orthographic Inconsistency in Reading: Evidence from a Cross-Linguistic Comparison

**DOI:** 10.1371/journal.pone.0157457

**Published:** 2016-06-29

**Authors:** Chiara Valeria Marinelli, Cristina Romani, Cristina Burani, Victoria A. McGowan, Pierluigi Zoccolotti

**Affiliations:** 1 Lab of Applied Psychology and Intervention, Department of History Society and Human Studies, University of Salento, Salento, Italy; 2 Neuropsychological Research Centre, IRCCS Fondazione Santa Lucia, Rome, Italy; 3 Aston University, Birmingham, United Kingdom; 4 ISTC Institute for Cognitive Sciences and Technologies, CNR, Rome, Italy; 5 Department of Life Sciences, University of Trieste, Trieste, Italy; 6 University of Leicester, Leicester, United Kingdom; 7 Department of Psychology, University of Rome La Sapienza, Rome, Italy; University of Valencia, SPAIN

## Abstract

We compared reading acquisition in English and Italian children up to late primary school analyzing RTs and errors as a function of various psycholinguistic variables and changes due to experience. Our results show that reading becomes progressively more reliant on larger processing units with age, but that this is modulated by consistency of the language. In English, an inconsistent orthography, reliance on larger units occurs earlier on and it is demonstrated by faster RTs, a stronger effect of lexical variables and lack of length effect (by fifth grade). However, not all English children are able to master this mode of processing yielding larger inter-individual variability. In Italian, a consistent orthography, reliance on larger units occurs later and it is less pronounced. This is demonstrated by larger length effects which remain significant even in older children and by larger effects of a global factor (related to speed of orthographic decoding) explaining changes of performance across ages. Our results show the importance of considering not only overall performance, but inter-individual variability and variability between conditions when interpreting cross-linguistic differences.

## Introduction

The present study regards reading acquisition in English and Italian, two languages with strong differences in orthographic regularity. Orthographic consistency is a major factor in how easily a child can learn to read [1]. This is clear from a large cross-linguistic investigation of 14 European languages in which word and non-word reading were measured at the end of first grade [2]. In word reading, accuracy was near ceiling in most languages with the most regular orthographies (*e*.*g*., German, Greek and Italian, one of the two languages investigated in the present study); it was lower (around 80%) in less consistent orthographies (*e*.*g*., French and Danish), and only 34% (over three SDs below the 14-nation mean) in English, the least regular orthography (the second language investigated in the present study). English children also had great difficulty reading non-words (29% accuracy compared to the cross-national average of 82%) [2].

These findings have been replicated in a number of smaller-scale studies that compared English-speaking children with children speaking German [3, 4], Dutch [5], Spanish [6], Portuguese [7], Italian [8], Greek [9], and Turkish [10]. Therefore, reading acquisition is faster in readers of regular orthographies during the first years of schooling. In irregular orthographies, beginning readers need a relatively long period of time to acquire and automatize irregular orthography-phonology mappings [11].

The fact that orthographic consistency has a striking effect at the beginning of learning does not necessarily indicate a difference at later phases of literacy acquisition. When tested with strictly matched stimuli, German and English speaking young adults performed similarly in terms of accuracy, although English adults tended to be faster [12]. Nevertheless, qualitative differences emerged: English speaking readers were more sensitive to body-rhyme effects (i.e., were facilitated in reading words that shared the orthographic rhyme such as street, meet, feet [12]) while German readers were more sensitive to length effects. As most studies examined early phases of learning to read, relatively little is known about the role of orthographic consistency in intermediate stages of literacy acquisition (such as late elementary school or middle school) and the few reported results are inconsistent. Patel, Snowling and de Jong [13] found smaller between-language differences in younger (eight-year-old) than in older (ten-year-old) children. Instead, two studies that examined children living in North Wales taught with similar methods, but with formal instruction in either English or Welsh (a consistent orthography), reported opposite results. There were larger differences when the children were younger (*i*.*e*., from five to seven years old [14]), but they reduced in older children [15], because children learning English caught up with those learning Welsh. Some differences, however, remained. The English children were faster in reading a text, but they were less accurate in reading low- and medium-frequency irregular words.

Most reviewed studies focussed on quantitative differences such as number of errors across orthographies; only a few (e.g., [16]) examined the influence of psycholinguistic variables (*i*.*e*., frequency, length, *etc*.) on reading development. Since orthographic consistency can influence the procedure or units of analysis used in reading as well as the ease of reading acquisition, examining psycholinguistic effects may be informative. In regular orthographies, learning to read may depend on qualitatively different mechanisms than those used in irregular orthographies [17]. Some differences have been reported in young readers. The length effect is greater in languages with more regular orthographies. Word length determined 70% of the difference in times to read words in Welsh, but only 22% in English [18]. In reading non-words, readers of regular orthographies display more exhaustive (and often laborious) letter-by-letter decoding than English readers (e.g., [19]). Moreover, English speaking readers make more real-word substitutions than readers of regular orthographies [2,4,14,18,19]. Overall, these findings indicate greater reliance on larger units of orthography-to-phonology conversion in inconsistent orthographies, at least in the early phases of literacy acquisition.

The present study investigated the reading skills of Italian and English children at two points in development (*i*.*e*., the middle and the end of elementary school) after basic literacy skills had been acquired to determine whether the orthographic characteristics of these two very different languages influence the reading profile. Our working hypothesis was that, in inconsistent orthographies, children are induced to use larger units of analysis because they are more reliable than smaller units [1,11]. We also investigated whether reading behaviour, as well as related cross-linguistic differences, may vary with reading experience. As mentioned, previous research yielded mixed results: Patel *et al*. [13] found larger cross-linguistic differences in older children while Hanley *et al*. [15] found the reverse pattern. However, cross-linguistic differences may vary as a function of age not only from a quantitative perspective, but also from a qualitative one. The high level of difficulty of the English orthography (due to the inconsistency of the grapheme-to-phoneme mappings and the high number of irregular words) would lead to worse performance in younger children. More qualitative differences, however, may emerge in older children. Both Italian and English children should improve their reading skills with age and reach similar general levels of performance. However, greater exposure to consistent/inconsistent orthographic patterns may lead older children to rely on different reading strategies, with greater reliance on larger units of analysis in children learning to read in English.

There are several problems in implementing cross-linguistic studies of reading acquisition. First, countries often differ with regard to school systems and have divergent curricula, teaching methods and different ages at which instruction begins. In Italy, formal reading instruction starts in first grade when children are, on average, six years old; in England, children start school at five years of age after a year of nursery transition during which they receive informal instruction about letter names and sight word reading. Moreover, different teaching methods are used for different languages and they may moderate or enhance cross-linguistic differences in reading development. However, in recent years [20] the instruction method used to teach English has become more similar to those used for transparent orthographies [19]. Moreover, language-specific differences have also been reported between children reading orthographies with different consistencies even though they live in the same area and follow similar curricula [14,18,19,21].

A second difficulty concerns matching stimuli across languages in terms of psycholinguistic variables. To understand commonalities and differences it is crucial to have comparable orthographic materials. Nevertheless, it may be difficult to select stimuli with similar psycholinguistic characteristics (*i*.*e*., frequency, regularity, syllabic structure, length, *etc*.) because of differences across languages. For example, studies usually report lower accuracy in inconsistent orthographies. However, here lists of words may include more irregular (hence more difficult) words. Thus, it may be difficult to identify a sufficiently large number of regular words when controlling for relevant linguistic parameters (i.e., number of letters, word frequency, *etc*.). Furthermore, while most English words are monosyllabic, languages with regular orthographies, such as Finnish or Italian, have many more long, polysyllabic words. More than 40% of Italian words are at least four syllables long, and morphologically complex words account for *ca*. 70% of low-frequency words [22].

A third problem is methodological. To compare languages, it is important to select stimuli that are not too easy or too difficult for the children examined. In transparent orthographies, however, accuracy asymptotes quickly in development and speed measures becomes preferable [17, 23]. Due to accuracy ceiling effects in regular orthographies, *ad hoc* “difficult” tasks may be used to detect individual variability. Alternatively, cross-linguistic comparisons may focus on speed measures which are open scale and less sensitive to the distortions typical of closed scales. Vocal reaction times (RTs) are widely used. They guarantee high sensitivity, but require a minimum level of accuracy (which may not always be ensured in younger children). Thus, only a few developmental cross-linguistic studies have compared onset RTs. Ellis and Hooper [18] and Hanley *et al*. [15] found that word reading was slower in Welsh than in English children. Similarly, Ellis *et al*. [16] found that children reading the two most regular orthographies (Greek and Hiragana) included in the study had longer RTs than English children. By contrast, Patel *et al*. [13] found that English children were slower than Dutch children in reading both words and non-words, with large differences especially for non-words in older children. Therefore, the few available studies do not provide results in a consistent direction and this may be due to factors such as lack of matching for length which has a different effect in orthographies with different levels of regularity [16,18].

A fourth, general problem in developmental studies, is that general levels of performance vary greatly with age. If any two groups (*e*.*g*., young adults and elderly) vary in general speed of processing (or global factor), differences depend on both the difficulty of a given task and the general differences in processing speed. This produces larger differences in more difficult conditions over and above the influence of the specific experimental manipulation, i.e., an “over-additivity” effect [24]. The presence of over-additivity may overestimate or underestimate the contribution of specific variables modulating reading performance. Therefore, it is important to control for over-additivity when comparing two languages particularly in the case of children of different ages. Faust *et al*. (1999) proposed the rate-and-amount model (RAM) to separate global and specific performance effects. Additionally, the difference engine model (DEM) focuses on the description of the role of global components of performance in speeded cognition and can be seen as an integration of the RAM for describing global factors [25]. We will refer to both models in interpreting the global components in our data. Faust *et al*. [24] also proposed a number of data transformations that allow controlling for the influence of the global factor in the data (*i*.*e*., the over-additivity effect) and reliably establishing the possible residual role of specific factors. Using this approach to study reading acquisition in Italian children, we found that changes in reading ability as a function of age reflected both the influence of specific factors (*i*.*e*., length, frequency, and lexicality) and of a global factor in information processing [23]. This factor was interpreted as indicating proficiency in orthographic decoding.

In the present study, we compared reading acquisition in Italian- and English-speaking elementary school children. In planning the study, we took particular care to control for all the above-described methodological concerns. As formal instruction begins at different ages in Italy and England, we matched children for both age and years of schooling. We considered two developmental stages: The “younger” children (about 7.5 years old) were in third grade in England and second grade in Italy and the “older” children (about 9.5 years old) were in fifth grade in England and fourth grade in Italy. We also tested Italian fifth graders to disentangle the influence of school attendance from chronological age. Note that we were interested in studying cross-linguistic differences/similarities in reading profile as a function of reading experience, but only when literacy was advanced. For this reason, we chose children who were about 7.5 years old in both languages. In fact, there is evidence that this is the age at which basic literacy acquisition is generally completed across languages (e.g., [26]). Both reading accuracy and vocal reaction times (RT) were examined to have sensitive measures for detecting cross-linguistic differences and similarities. We generated lists of words (varying for frequency and length) and non-words (varying for length), matched for as many other psycholinguistic variables as possible. Note that only words with regular mapping in Italian and English were included to ensure that cross-linguistic differences in the reading strategy used were not due to the presence of a higher number of inconsistent words in English. To evaluate general changes in orthographic decoding skill with age and orthography reading data were analyzed with reference to the RAM [24] and DEM [25] models. This allowed establishing the specific influence of the frequency, lexicality and length effects on the reading performance of English and Italian children controlling for over-additivity effects.

Our analyses had three general aims:

1. to assess the influence of psycholinguistic variables (length, frequency and lexicality) on reading speed and accuracy and possible differences across languages and ages;
2. to establish in both languages whether (and in what measure) a *global factor* could account for the differences in reading speed between types of words and participants of different ages. To do this, we referred to two models that make different (but integrative) predictions to evaluate global factors: the RAM [24] and the DEM [25];
3. to establish whether the effects of psycholinguistic variables changed across languages and ages, when the effect of the global factor was taken into account.

Our general expectation was that, due to orthographic inconsistencies, the English language would produce greater reliance on larger units of analysis than the Italian language. Related to this, we also expected: (a) greater effects of frequency in younger English readers than in Italian readers, because lexical processing is more important for correct and fast reading in English even in the early stages of learning to read; (b) smaller length effects in English than Italian readers.

## Methods

### 1 Participants

The following inclusion criteria were used to select the two (English and Italian) samples: (a) growing up in a social and educational context with adequate literacy opportunities. We did not include foreign children and children with cognitive impairment (who performed below 1.5 standard deviations at the Raven’s CPM [27]). Children were selected from local public primary schools (in England in the Birmingham area and in Italy in the Rome and Naples areas). Parents were informed about the screening activities and authorized their child’s participation by signing the appropriate informed consent paperwork. The study was conducted according to the principles of the Helsinki Declaration and was approved by the local committee of the Departments and by the school authorities. The study was reviewed and approved by Aston University Research Committee and by the Ethics Committee of the IRCCS Fondazione Santa Lucia Rome (Prot. CE-PROG.480) before the study began.

The subgroups matched for age included a total of 177 Italian children (87F, 90M) and 81 English children (43F, 28M). There were 90 younger children in Italy (43F, 47M, mean age = 7.3 years) and 40 in England (17F, 23 M, mean age = 7.8 years). There were 87 older children in Italy (44F, 43 M, mean age = 9.6 years) and 41 in England (26F, 15M, mean age = 9.9 year). Younger children were in third grade in England and second grade in Italy; older children were in fifth grade in England and fourth grade in Italy. Matched groups did not differ for gender (all *χ*^2^ about 1). A quantitatively small, but significant difference was present for age (for younger children: *t* (128) = 11.52, p < .0001; for older children: *t* (126) = 5.91, p < .0001); the English children were ca. four months older than the Italian children. Note that in England children are admitted to first grade if they have reached the appropriate age within August-September, whereas in Italy this limit is delayed until December. This difference is presumably responsible for the difference in the ages of the Italian and English samples. Performance on Raven’s test was better in English children (younger children: *t* (128) = 3.51, p < .0001; older children: *t* (126) = 3.16, p < .01) than in Italian ones.

An additional group of 30 Italian (17F, 13M) fifth graders (mean age = 10.6 year) was matched with the fifth grade English children for number of years of schooling. The two fifth grade groups did not differ for gender (*χ*^2^ < 1) or Raven performance (*t* < 1), but differed for age (*t* (68) = 7.86, p < .0001); the Italian children were older by ca. seven months.

### 2 Experimental task

#### 2.1 Materials

We used a list of 120 stimuli for each language (Italian and English; see Appendix). The list served to assess the effects of length (4, 5, 6 and 7–9 letters) and stimulus type (high frequency words, low frequency words and non-words), for a total of ten stimuli in each sub-set. Only Italian words with regular stress (*i*.*e*., on the penultimate syllable) and English words with regular correspondences (no letter-sound correspondence with a frequency of less than 5% according to Hanna et al. [28]) were included in the word sets. High frequency words had a mean frequency of 106.2 (SD = 94.2) in English (CELEX lexical database, [29]) and 63.7 (SD = 55.6) in Italian (CoLFIS database, [30]); low frequency words had a mean frequency of 2.9 (SD = 1.4) in English and 3.2 (SD = 3.2) in Italian. Both Italian and English word frequencies were calculated out of 1,000,000 occurrences. The sets were balanced for ortho-syllabic difficulty (presence of double consonants, clusters of consonants and contextual rules [31]), articulation point of the first phoneme, and word frequency, but not for the number of syllables that was systematically higher in the Italian sets (see Appendix).

Non-words were created from high frequency words by changing one to three letters. Non-words had the same ortho-syllabic difficulty as words (presence of double consonants, clusters of consonants and contextual rules, bigram frequency, *etc*.).

#### 2.2 Procedure

Children were tested individually in a quiet room at their school using a portable computer. They were seated ca. 60 cm from the computer screen. Stimuli were presented using the E-prime2 software. Each trial began with a fixation point that remained on the screen for 500 ms. Subsequently, a word appeared in the same position. The stimulus remained on the screen until the child responded.

Words and non-words were presented in separate blocks. To make the task less tiring, words were divided into three separate blocks with a brief pause between them. To avoid the priming of non-words by the words they were derived from, the non-word block was presented before the word blocks. Six practice stimuli preceded both the word and non-word reading trials. In both words and non words blocks, the order of trials presentation was randomized for each child.

The child was requested to read the stimulus as quickly and accurately as possible. Vocal RTs were recorded using a voice key (S-R Box). The experimenter manually recorded pronunciation errors. The responses were tape-recorded to allow offline rechecking. The RTs corresponding to errors were excluded from the analyses. Self-corrections and wavers were considered errors and the corresponding RTs were not included in the analyses. Invalid responses (due to technical problems) and RTs below 250 ms or exceeding the individual mean ± 3 standard deviations were also excluded from the analyses. There were few invalid RTs in both the Italian (second grade: 2.2%, fourth grade: 3.7%, fifth grade: 2.3%) and the English (third grade: 2.4%, fifth grade: 3.3%) samples.

### 3 Data analysis

The presence of a global factor accounting for some of the differences in reading between types of words and participants of different ages can be reliably detected for open scale scores, such as RTs, but not for closed scale scores, such as accuracy scores (because of the possible confound between ceiling/floor effects and over-additive effects). The presence of a global factor in each language can be established if the following conditions are present:

1. According to the RAM [24], there should be a linear relationship between the condition means of two groups that vary in overall information-processing rate, *i*.*e*., younger and older children. Thus, we plotted the mean RTs for all types of stimuli across younger and older children of each language. We expected differences between younger and older children to increase linearly with difficulty of the condition (the more difficult the words, the larger the difference).
2. According to the DEM [25], there should be a linear relationship between the group means and the standard deviations in the same conditions. The more difficult conditions should yield greater individual variability over and above the role of specific experimental manipulations (e.g., there should be a larger difference between good and poor readers in reading difficult words). It is worth noting that standard parametric analyses assume that variance is homogeneous across experimental conditions; thus, the presence of a significant relationship between SDs and the corresponding condition means represents a systematic deviation from this assumption. To test this prediction, we plotted mean RTs for all types of stimuli as a function of the corresponding standard deviations. According to the DEM model, the x-intercept allows estimating the sensory-motor component of the response, *i*.*e*., the time required for early visual processing, response selection and execution. We created different plots for the four groups of participants (i.e., younger and older children who spoke English or Italian), as recommended by Myerson *et al*. [25]. The slope of the regression and the intercept of the x-axis were predicted to be constant across different groups of children.

Once the presence of global factors was established for the two languages, we removed their effect from the effect of different psycholinguistic variables. This was made possible by analyzing transformed z-score data, which represent the deviation of each condition from the overall participant’s mean (subtracting the mean of each condition from the overall participant mean and dividing the product by the standard deviation of the condition means for each child). This transformation allows controlling for global components, but preserves the individual variability across experimental conditions. We carried out ANOVAs assessing the effects of the psycholinguistic variables in relation to language and age of participants on both raw RTs and z-transformed RTs. The effects that remained significant when the presence of a global factor was accounted for (*i*.*e*., in the z-transformed data) highlighted a genuine effect; effects that were significant only in the raw data, not in the z-transformed values, indicated the presence of a spurious interaction (due to the presence of over-additivity) [24]. Although not explicitly predicted by the RAM, the opposite pattern may also occur; *i*.*e*., an interaction may be significant in the z-transformed, but not in the raw data analysis. Controlling for the influence of global components may enhance the sensitivity of statistical comparisons and allow detecting differences that are masked in the raw data analyses. Separate analyses were carried out to compare groups matched for age and groups matched for school year. The variables entered in the analyses are presented in the Results section.

To control for non-linearity in the distribution of accuracy results and for possible floor effects, analyses on errors were carried out by means of Generalized Linear Mixed Models for binomially distributed outcomes [32]. The dependent variable was accuracy in reading each stimulus (1 = passed, 0 = failed) and the various effects (see Results section) were entered as fixed factors. However, for the sake of presentation, Tables 1 and 4 report the means of the percentages of errors.

**Table 1:** Means (and standard deviations) of RTs and percentages of errors in reading the different types of stimuli collapsed for length, for each group of children.

	YOUNGER CHILDREN		OLDER CHILDREN		
	Italian	English	Italian	English	Italian
Mean years of age	7–8	7–8	9–10	9–10	10–11
Grade	2nd	3rd	4th	5th	5th
RTs (ms.)					
Non words	2099 (1503)	1687 (1194)	1391 (666)	1225 (862)	1213 (597)
Hf words	1372 (829)	1121 (988)	889 (342)	805 (609)	861 (412)
Lf words	1520 (809)	1345 (1055)	1043 (435)	948 (798)	957 (504)
Errors (%)					
Non words	25.0 (16)	26.4 (22)	10.4 (9)	14.1 (14)	12.1 (12)
Hf words	7.8 (10)	6.2 (11)	1.6 (3)	4.5 (9)	1.8 (5)
Lf words	14.7 (13)	17.9 (19)	4.5 (5)	6.4 (11)	4.5 (8)

Note: Hf = High frequency; Lf = Low frequency

## Results

**Fig 1:**
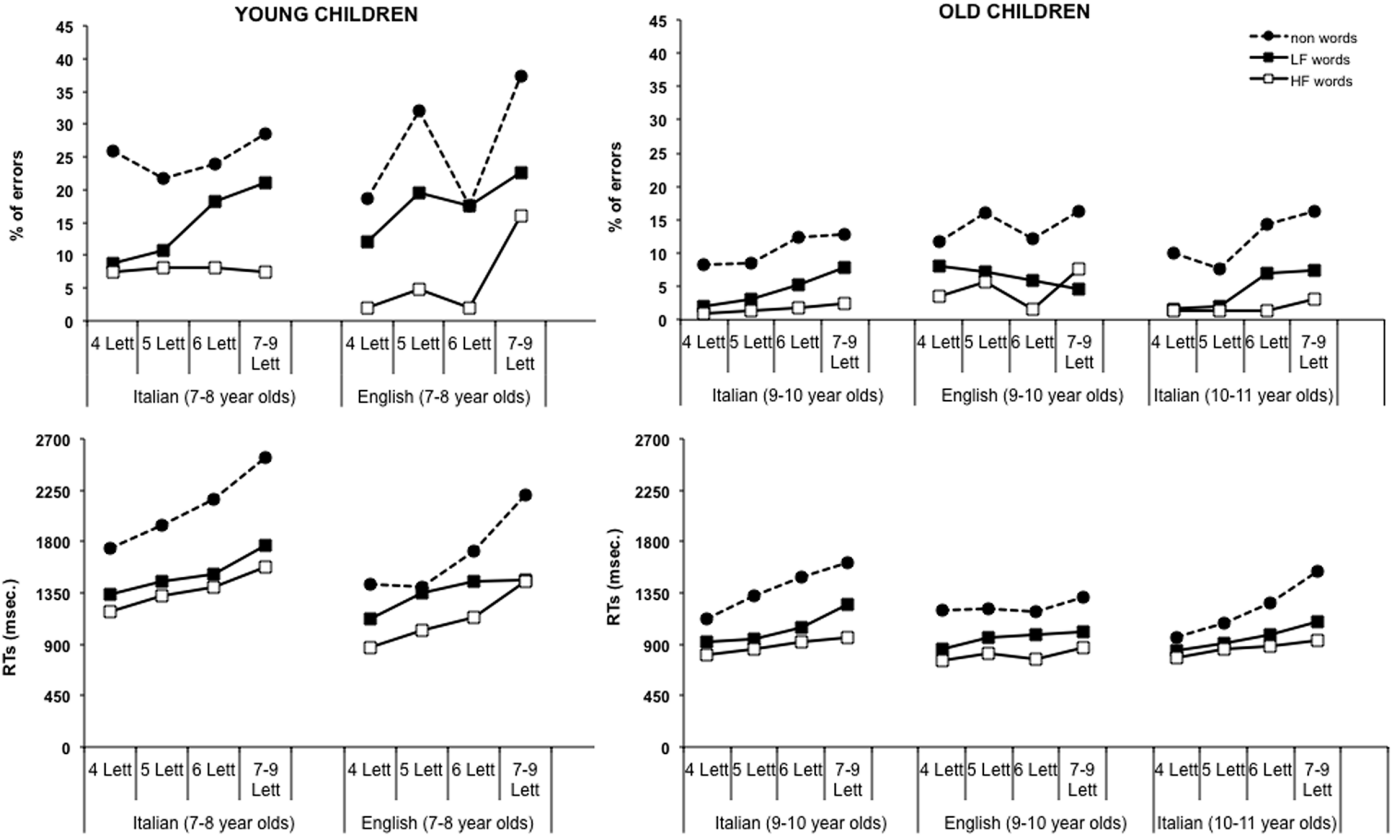
Mean percentages of errors (upper part) and RTs (bottom part) in reading different types of stimuli as a function of language and grade. The figure shows the effects of lexicality and frequency as a function of length of the stimuli (from 4- to 7-9- letters).

Table 1 reports the mean RTs and errors in reading words and non-words for each group of English and Italian children (note that we make the raw data accessible upon request to all interested researchers). Fig 1 illustrates the main data (RTs and reading errors) of the main conditions, separately for the various subgroups. An inspection of these plots allows for a number of general observations:

- in both languages children’s performance (*i*.*e*., RTs and errors) improves substantially with age/reading experience;
- as a group English children tend to read faster but also to make more errors than Italian children (see Table 1). These differences are relatively small and vary across conditions
- as to accuracy, some conditions (particularly non-words and long LF words) are associated with large proportions of errors among younger children; however, there seems to be no sign of a speed-accuracy trade-off.
- in English, HF 6-letter words (and derived non-words) generate fewer errors than stimuli of other lengths (a tendency not present with RTs), presumably because several stimuli in these two sets ended in–er. It has been reported that both words and non-words ending in -er are read more accurately by English speaking children than those having different endings [33];
- length effects are present in younger children in both languages; in older children, they are more apparent in Italian than English children (irrespective of stimulus type);
- frequency, lexicality and regularity effects were all detectable, although varied across ages and language groups.

### 1 Detecting global components in the data

**Fig 2:**
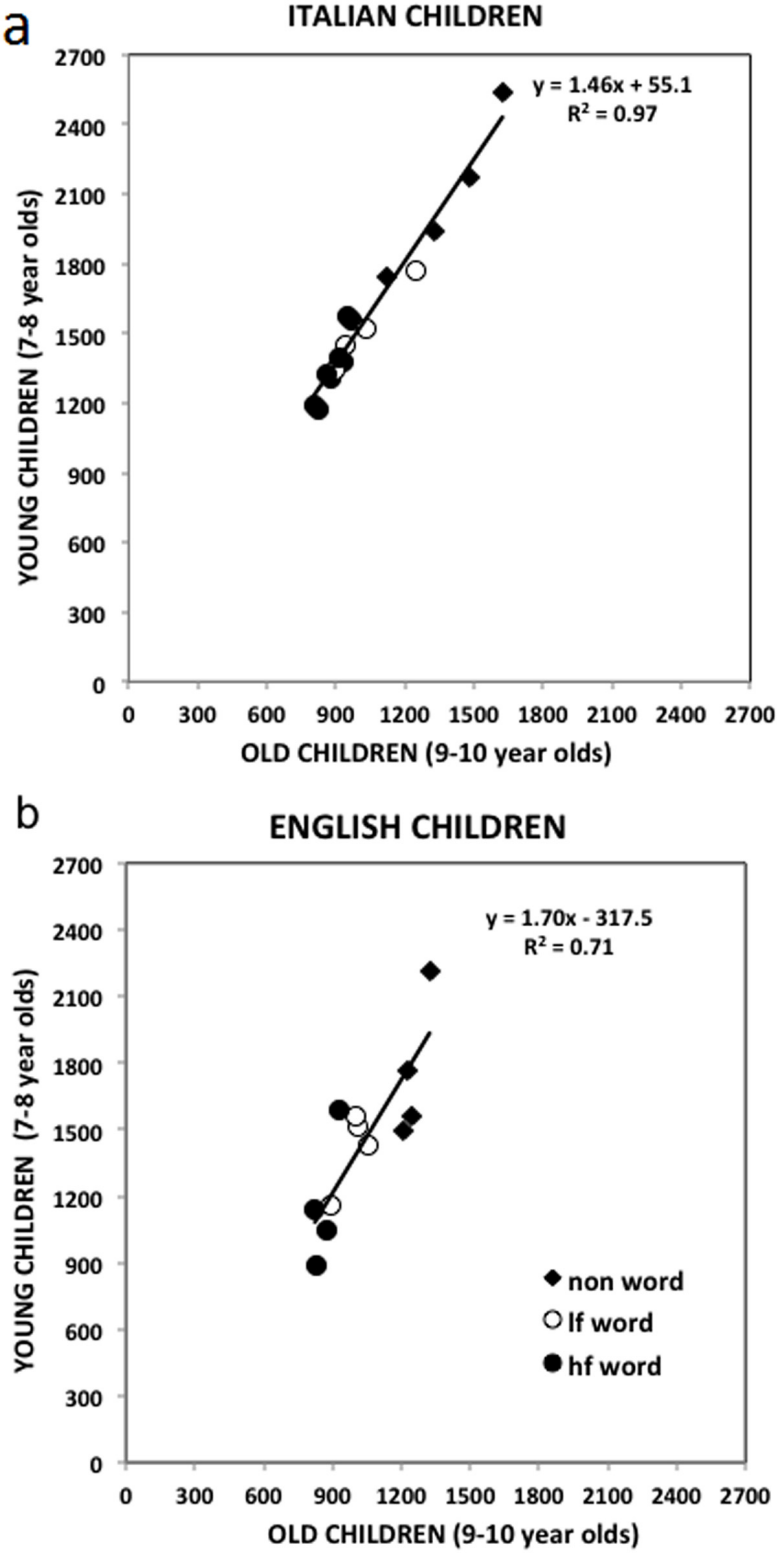
Condition means of younger children are plotted as a function of older readers’ means in English (Fig 2a) and Italian (Fig 2b) children. The regression lines and proportions of explained variance are also reported in the graphs. The diagonal line (slope = 1) represents equal RTs for younger and older children. Legend: lf word = low frequency words; hf word = high frequency words. For each experimental condition represented in the plot with a different symbol we have four points, corresponding to the four length data points (4-, 5-, 6-, 7/9- letters nonwords/low frequency words/high frequency words, respectively).

Younger and older children’s condition means are plotted against each other in Fig 2a (Italian children) and 2b (English children). The diagonal dotted line in the graph indicates the reference for identical performance of the two groups.

Fig 2a shows that a single regression line interpolates the Italian data extremely well and explains a very large proportion of variance (*r*^*2*^ = .97). Conditions varied greatly (reflecting the influence of lexicality, frequency and length), but all contributed to the same global factor. The older children were faster and this difference was larger for the more difficult conditions (the slope was steeper than the reference slope). The older children read 46% faster than the children who were two years younger (as indicated by a slope of *b* = 1.46). Fig 2b shows that a single regression line also interpolates the English data relatively well, indicating the presence of a global factor. However, this factor explains a smaller proportion of variance (*r*^*2*^ = .71), as indicated by the larger spread in the plot. Therefore, different from the Italian children, the improved performance of English children with age/reading experience was not entirely explained by a single global factor. Overall, the older English children read 70% faster than children who were two years younger (*b* = 1.70).

**Fig 3:**
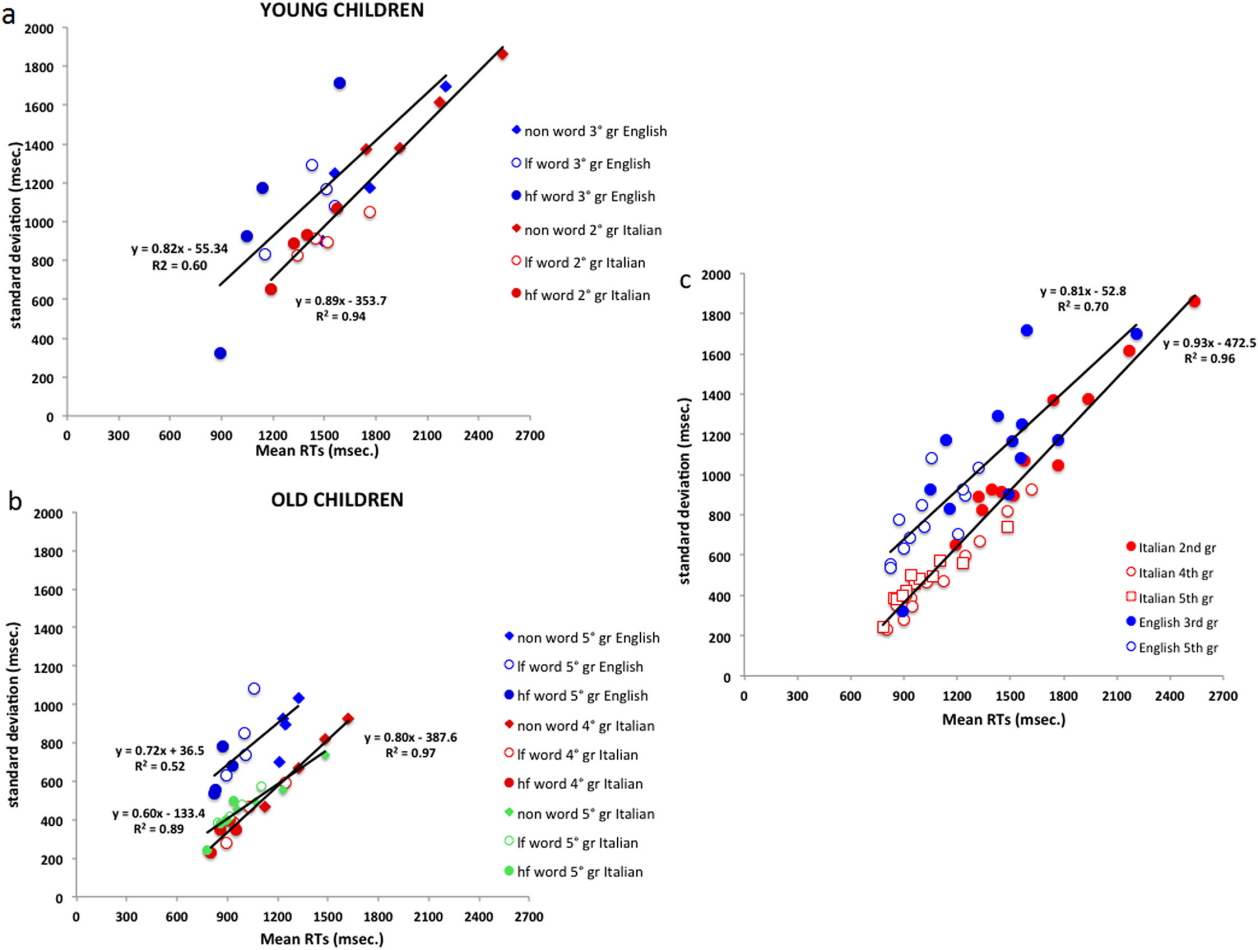
Standard deviations across individuals are plotted as a function of overall group RT means for the same conditions, in younger (Fig 3a) and older (Fig 3b) Italian and English children. Plot C reports together the RT means and standard deviations of each group of Italian and English children. Legend: lf word = low frequency words; hf word = high frequency words. Again, the four data points for each experimental condition correspond to the four lengths.

Fig 3a and 3b plot condition means against the standard deviations of the same conditions for the younger and older samples, respectively. Data on both Italian and English children are shown jointly in the same figure to highlight the comparison between the two languages. In Fig 3c, the same data are plotted by presenting younger and older children together to highlight the effects of developmental stage.

Fig 3a shows that a single regression line accounts extremely well for the Italian data, indicating a steady increase in variability in progressively more difficult conditions (*r*^2^ = .94); the same relationship is present, but less clear, in the English sample (*r*^2^ = .60). The slope of the two regression lines is approximately the same in the two languages (.89 in the Italian sample and .82 in the English sample, respectively). The intercept on the abscissa (which, based on DEM, represents an estimate of the sensory-motor component of the response), is smaller for the English third graders (68 ms) than the Italian second graders (399 ms). Also, the English children tend to be faster (*i*.*e*., their data points tend to be on the left of those of the Italian children on the abscissa). The English children also show more inter-individual variability within conditions (*i*.*e*., larger SDs per condition), as indicated by the higher data points of the English children on the ordinate than the Italian children.

Fig 3b shows a similar pattern in older readers. A single regression line accounts extremely well for the Italian data (*r*^2^ = .97); a similar relationship is present, but less clear, in the English sample (*r*^2^ = .52). The slope of the two regression lines is .80 for the Italian sample and .72 for the English sample. The intercept on the abscissa is smaller (and actually negative) for the English (-51 ms) than the Italian (482 ms) sample. Data for the Italian fifth graders are generally similar to those of the Italian fourth graders: The slope is .60 and the intercept 222 ms (*r*^2^ = .89). Again, the English children are faster than the Italian children and show more inter-individual variability in all conditions (larger SDs). However, the older English children also show a smaller range of performance between conditions with less of a spread on the abscissa. In other words, there is a greater difference between fast and slow children, but there is less difference across conditions (less differences in frequency, length and lexicality).

Fig 3c shows that the association between level of performance (speed) and variability (SD) is the same for faster and slower individuals (here, older and younger children, respectively) and across the two languages, consistent with the prediction of the DEM. In fact, the slopes for the two languages are quite similar; they differ primarily because of a larger intercept in the Italian sample. However, this figure shows again that the English sample is characterized by larger inter-individual variability (higher SDs per condition) and a smaller spread across conditions. *Ad-hoc* analyses were carried out to clarify this difference in individual variability.

### 2 Analyzing individual differences across languages

We examined two possible explanations for the greater variability in the performance of the English children.

First, we investigated whether the English children were also more variable within participants with increased variability between different trials. We plotted the mean RTs of each child in each condition against the standard deviation of the same condition to check whether the languages differed due to the variability produced by the items to be read. These analyses yielded a large number of plots (one for each condition in each age/language group). A synthesis of these analyses is presented in Supporting Information file (S1 Table). Overall, we observed very similar relationships across ages and languages. For each condition, and across languages, the individual standard deviation was predicted by the mean RTs. The faster children were also the least variable, as expected. Therefore, these data indicate that the large variability observed in the English children was not due to a general increase in variability in the English data.

**Fig 4:**
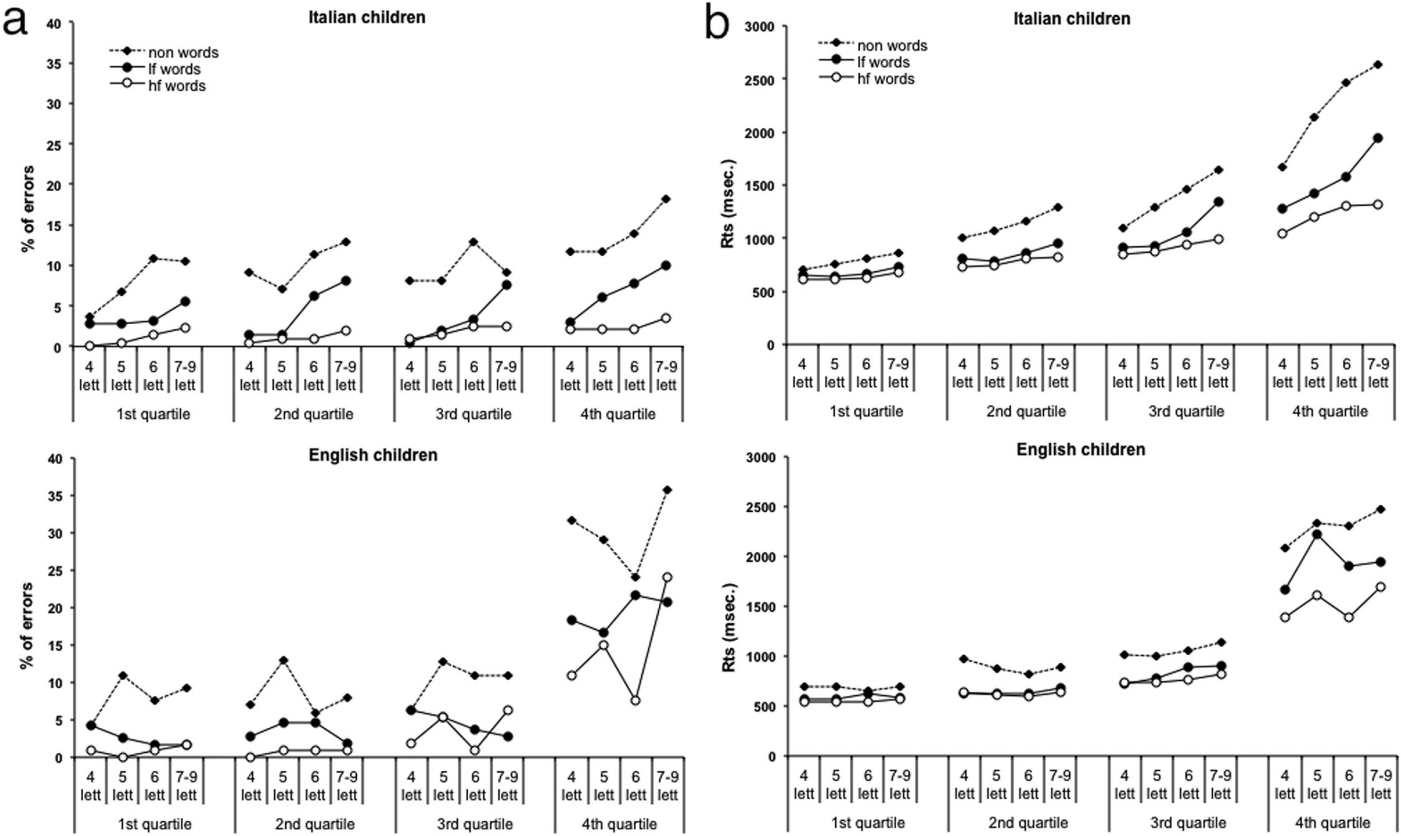
Performance of 9–10 year-old Italian and English children separated into four quartiles according to their reading performance. Figures A and B report the mean percentages of errors and the RTs of the four quartiles in each experimental condition, respectively. Legend: lf word = low frequency words; hf word = high frequency words; lett = letters.

**Fig 5:**
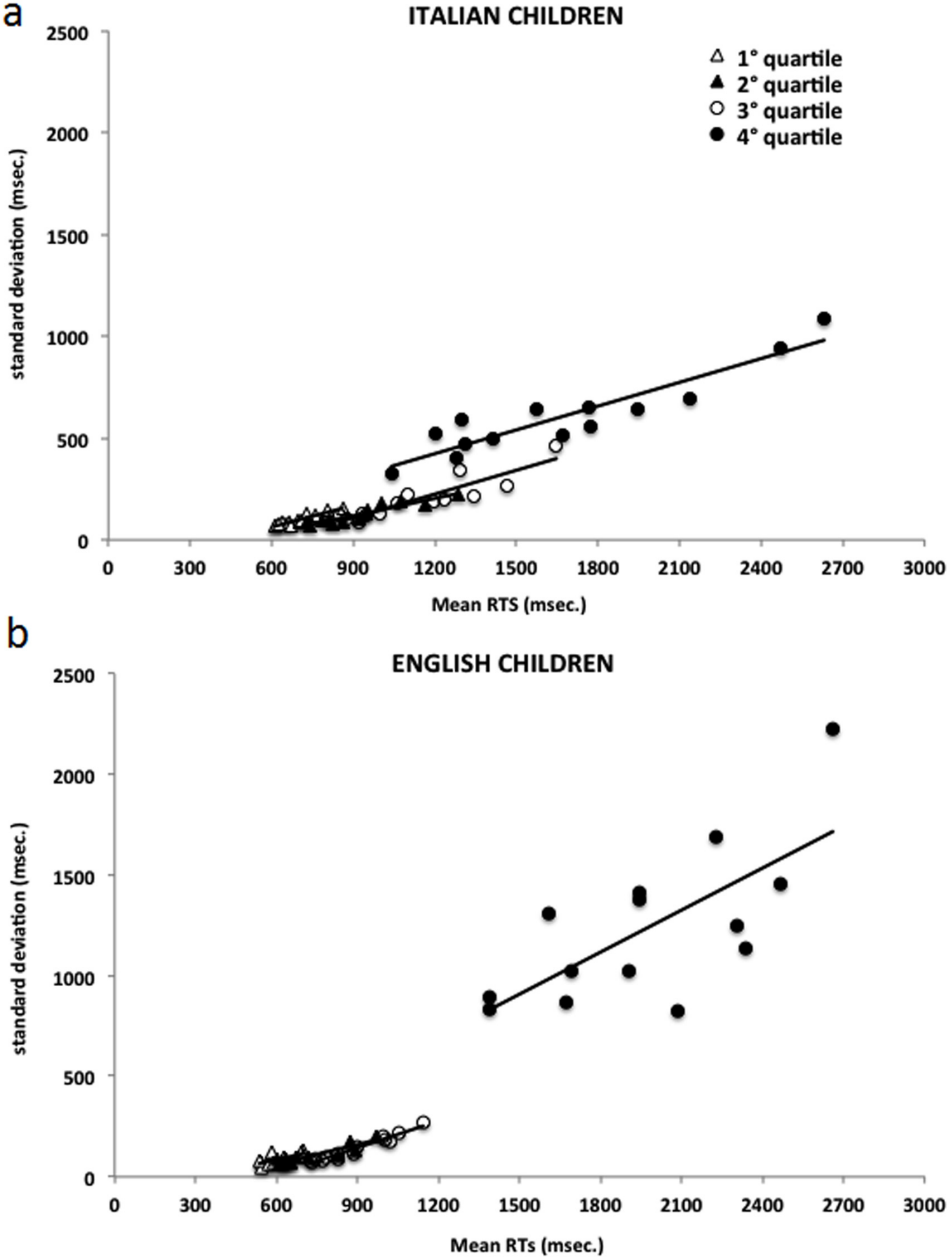
The standard deviations are plotted as a function of the means for the same conditions for each quartile. Legend: lf word = low frequency words; hf word = high frequency words; lett = letters.

Second, we examined inter-individual variability by dividing the children of each language into approximate quartiles based on overall RTs across conditions. For the sake of brevity, to illustrate this pattern of results we compare Italian fourth graders and English fifth graders. Errors and RTs in all conditions are reported in Fig 4a and 4b. In the English children, the first three groups differ relatively little: their RTs are relatively fast and vary little with word length. By contrast, the fourth quartile shows much slower RTs and many more errors as well as large effects of frequency, lexicality and length. In the Italian children, instead, there is a much smoother decrease in performance from the first to the fourth quartile for both RTs and errors. Fig 5 plots the condition means against the standard deviations of the same conditions for the four quartiles of the Italian (a) and English (b) children. For the English children, the first three quartiles are very similar but the fourth quartile stands out, showing no overlap with the other three. Note that the fourth quartile presents a large spread among conditions as well as large SDs and a large deviance from the prediction based on a global factor (including a steeper slope than the other three quartiles). The Italian children, by contrast, show more overlap between quartiles. Furthermore, slopes and intercepts are quite similar among the four groups, as predicted by the DEM [25]. By definition the children in the fourth quartile are slower, but their reading performance did not deviate qualitatively from that of the children in the other groups.

#### 2.1 Summary and comments

The influence of a global factor was detected for both English and Italian children. This factor explained part of the performance for all types of words and non-words: reading performance improved with increasing age, largely independently of the stimulus type. Furthermore, the plots indicated a linear relationship between condition means and standard deviations. As predicted, slower, more difficult conditions showed more inter-individual variability.

Our results also indicate important differences between the two languages. First, the global factor explains a very large proportion of variance in the Italian data and a smaller one in the English data. In the English children, the increase in performance with age is not entirely explained by the overall improvement in information-processing rate. The ANOVAs on RTs and z-transformed data presented in the following sections will provide a systematic evaluation of these specific influences. Second, with increasing age English children improved their reading speed more than Italian children (70% *vs* 46%). Third, the intercept on the x axis is approximately what we expected (*e*.*g*., 300 ms [according to 25]) in the Italian sample; this represents an estimate of the sensory-motor (peripheral) processes not correlated with the duration of the cognitive portion of that task. The intercept on the x axis is considerably smaller in the English samples (below 100 ms) or even negative in fifth graders. Therefore, the peripheral processes cannot be reliably estimated in English children. Fourth, the most substantial difference lies in the relationship between condition means and variability: English children are more variable than Italian children but are faster, not slower, as one would expect from the influence of a single global factor (which should yield larger variability values for more difficult conditions and/or slower individuals). Finally, particularly in older children, there is less difference between the easiest and most difficult condition in the English than the Italian sample. Overall, the English data are more variable across individuals and less variable across conditions. This pattern holds for both a chronological and a school match. The larger inter-individual variability in the English children is not due to a general increase in variability in the data. This is supported by the fact that the English children also do not show larger inter-trial variability.

We further explored inter-individual variability by looking at sub-groups of children that differed for reading speed. Italian children closely fit the predictions of a single global performance factor. Differences in performance as a function of reading proficiency or type of condition were systematically associated with differences in variability, with easier stimuli and older children showing less variability. The English data fit the predictions of a global factor less well. Most children showed fast RTs with a limited spread across conditions and limited individual differences. However, a substantial sub-group of children showed a deviant pattern in terms of RTs and errors. The conjunction of these characteristics produced the complex pattern described above, *i*.*e*., faster RTs in the presence of larger inter-individual variability and a small intercept on the x axis.

The present results are similar to those reported by Spencer and Hanley [14] and Hanley et al. [15]: the English children in their lowest quartile performed quite differently than those in the other three quartiles, an effect which was not present among children learning Welsh (a regular orthography). Note that, although both speed and accuracy were measured, the quartile analysis was restricted to accuracy measures [15].

The differences outlined above can be related to the differences in consistency between the two languages. In Italian words are read across ages relying on orthographic decoding. Orthographic decoding will be faster for short than long words and for high than low frequency words, but differences across ages can be largely explained in terms of increased proficiency with this procedure. In English, the situation is different: something else contributes to a) increased proficiency across ages, b) overall faster RT and increased variability across individuals. We will argue that this is due to reliance on larger processing units which is not uniformly acquired across ages and across individuals (more in the General Discussion).

### 3 Language comparison

We carried out two sets of analyses to examine the influence of language on *frequency* and *lexicality* effects (with each variable crossed with stimulus length). RTs were explored with ANOVAs and errors with Logistic Mixed Effect Models.

Firstly, we compared English and Italian children matched for age. In the first ANOVA, *frequency* (high, low) and *length* (4, 5, 6, 7–9 letters) were repeated measures, and *age* (7.5 years, 9.5 years) and *language* (English and Italian) were between-groups measures. In a second ANOVA, *lexicality* (words, non-words) and *length* were repeated measures and *age* and *language* were between-groups. Note that because non-words were generated from high frequency words, high frequency words were compared to non-words in the analysis of the lexicality effect. In a second set of analyses, we compared children matched for number of years of schooling (*i*.*e*., fifth grade Italian and English children). As above, separate ANOVAs were carried out with *frequency* and *length* and with *lexicality* and *length* as repeated measures. Interactions were explored with planned comparisons. We present RT analyses and z-transformed values jointly to show which interactions can be interpreted as over-additivity effects.

Errors were examined using Logistic Mixed Effect Model. A first analysis entered *frequency*, *length*, *age* and *language* as fixed factors and *items* and *participants* as random factors. A second analysis entered *lexicality*, *length*, *age* and *language* as fixed factors, while *items* and *participants* were random factors. The same analyses were run also on fifth grade children only: in this case only *length* and *language* and *frequency* or *lexicality* were entered as fixed factors.

Results of analyses examining the effects of “*age*” and “*school-year”* are reported in Tables 2, 3, 4 and 5, respectively. In the former case, the two groups of children (Italian and English) were matched for age, independently of schooling level. In the latter case, analyses compared children matched for number of years of schooling. For the sake of simplicity, main effects and interactions are described as a function of raw RTs and percentage of errors.

**Table 2:** Results of the ANOVAs on reading RTs (both raw and Z transformed data) in the English-Italian comparisons (see text for details).

	RTs				
EFFECT	df	raw data		z score	
		F	p	F	P
**FIRST ANALYSIS: FREQUENCY EFFECT**					
**Language**	1,254	2.67	--	--	--
Age	1,254	20.34	<0.0001	--	--
Frequency	1,254	84.90	<0.0001	269.83	<0.0001
Length	3,762	54.49	<0.0001	167.18	<0.0001
**Language** by Age	1,254	0.44	--	--	--
Frequency by **Language**	1,254	0.80	--	0.33	--
Frequency by Age	1,254	1.03	--	0.11	--
Frequency by **Language** by Age	1,254	1.44	--	2.01	--
Length by **Language**	3,762	0.98	--	3.79	0.010
Length by Age	3,762	8.84	<0.0001	5.96	0.0005
Length by **Language** by Age	3,762	1.47	--	2.10	--
Frequency by Length	3,762	0.28	--	6.74	0.0002
Frequency by Length by **Language**	3,762	4.50	0.004	11.17	<0.0001
Frequency by Length by Age	3,762	2.62	0.049	0.95	--
Frequency by Length by Age by **Language**	3,762	0.36	--	1.42	--
**SECOND ANALYSIS: LEXICALITY EFFECT**					
**Language**	1,254	4.22	0.041	--	--
Age	1,254	19.60	<0.0001	--	--
Lexicality	1,254	104.35	<0.0001	1366.75	<0.0001
Length	3,762	85.10	<0.0001	192.10	<0.0001
**Language** by Age	1,254	0.86	--	--	--
Lexicality by **Language**	1,254	1.26	--	3.73	--
Lexicality by Age	1,254	2.92	--	16.71	<0.0001
Lexicality by **Language** by Age	1,254	0.13	--	1.60	--
Length by **Language**	3,762	2.16	--	13.17	<0.0001
Length by Age	3,762	23.44	<0.0001	15.87	<0.0001
Length by **Language** by Age	3,762	3.71	0.010	8.47	<0.0001
Lexicality by Length	3,762	1.94	<0.0001	17.07	<0.0001
Lexicality by Length by **Language**	3,762	2.94	0.032	4.75	0.003
Lexicality by Length by Age	3,762	1.85	--	3.75	0.020
Lexicality by Length by Age by **Language**	3,762	0.29	--	4.90	0.002

**Table 3:** Results of the Logistic Mixed Effect Model on accuracy data in the English-Italian comparisons (see text for details).

	% OF ERRORS		
EFFECT	df	Q	p
**FIRST ANALYSIS: FREQUENCY EFFECT**			
**Language**	1,20521	6.10	.014
Age	1,20521	326.55	<0.0001
Frequency	1,20521	152.90	<0.0001
Length	3,20521	29.64	<0.0001
**Language** by Age	1,20521	18.91	<0.0001
Frequency by **Language**	1,20521	5.16	0.023
Frequency by Age	1,20521	1.37	--
Frequency by **Language** by Age	1,20521	7.80	0.005
Length by **Language**	3,20521	6.88	<0.0001
Length by Age	3,20521	0.48	--
Length by **Language** by Age	3,20521	6.73	<0.0001
Frequency by Length	3,20521	4.58	0.003
Frequency by Length by **Language**	3,20521	8.62	<0.0001
Frequency by Length by Age	3,20521	0.37	--
Frequency by Length by Age by **Language**	3,20521	2.48	--
**SECOND ANALYSIS: LEXICALITY EFFECT**			
**Language**	1,20521	24.70	<0.0001
Age	1,20521	217.65	<0.0001
Lexicality	1,20521	569.53	<0.0001
Length	3,20521	31.56	<0.0001
**Language** by Age	1,20521	24.29	<0.0001
Lexicality by **Language**	1,20521	3.90	0.048
Lexicality by Age	1,20521	3.30	--
Lexicality by **Language** by Age	1,20521	4.53	0.033
Length by **Language**	3,20521	12.90	<0.0001
Length by Age	3,20521	0.88	--
Length by **Language** by Age	3,20521	6.26	<0.0001
Lexicality by Length	3,20521	2.76	0.041
Lexicality by Length by **Language**	3,20521	3.39	0.017
Lexicality by Length by Age	3,20521	0.26	--
Lexicality by Length by Age by **Language**	3,20521	4.69	0.003

Note that *Item* and *Participant* were entered as random effects in the Logistic Mixed Effect Model analysis on accuracy data. The effects of *item* and *participant* were not significant in the first analysis of the frequency effect as well in the second analysis of the lexicality effect (all Zs < 1).

**Table 4:** Results of the ANOVAs on reading RTs (both raw and Z transformed data) in the 5^th^ grade English-Italian comparisons (see text for details).

	RTS				
	df	Raw data		Z scores	
		F	p	F	P
**FIRST ANALYSIS: FREQUENCY EFFECT**					
**Language**	1,69	0.07	--	--	--
Frequency	1,69	21.61	<0.0001	76.51	<0.0001
Length	3,207	8.62	<0.0001	29.23	<0.0001
Frequency by **Language**	1,69	0.83	--	0.78	--
Length by **Language**	3,207	0.80	--	1.27	--
Frequency by Length	3,207	1.30	--	3.34	0.020
Frequency by Length by **Language**	3,207	0.62	--	4.67	0.004
**SECOND ANALYSIS: LEXICALITY EFFECT**					
**Language**	1,69	0.03	--	--	--
Lexicality	1,69	56.37	<0.0001	614.08	<0.0001
Length	3,207	25.70	<0.0001	62.11	<0.0001
Lexicality by **Language**	1,69	0.43	--	1.75	--
Length by **Language**	3,207	9.00	<0.0001	24.73	<0.0001
Lexicality by Length	3,207	6.56	0.0002	12.41	<0.0001
Lexicality by Length by **Language**	3,207	5.04	<0.002	19.87	<0.0001

**Table 5:** Results of the Logistic Mixed Effect Model on accuracy data in the 5^th^ grade English-Italian comparisons (see text for details).

	% OF ERRORS		
	df	Q	P
**FIRST ANALYSIS: FREQUENCY EFFECT**			
**Language**	1,5584	5.96	0.015
Frequency	1,5584	21.90	<0.0001
Length	3,5584	5.39	0.001
Frequency by **Language**	1,5584	0.43	--
Length by **Language**	3,5584	4.05	0.007
Frequency by Length	3,5584	2.49	--
Frequency by Length by **Language**	3,5584	0.68	--
**SECOND ANALYSIS: LEXICALITY EFFECT**			
**Language**	1,5584	4.18	0.041
Lexicality	3,5584	111.64	<0.0001
Length	1,5584	3.07	0.027
Lexicality by **Language**	3,5584	0.08	--
Length by **Language**	3,5584	2.11	--
Lexicality by Length	3,5584	0.54	--
Lexicality by Length by **Language**	1,5584	0.17	--

Note that *Item* and *Participant* were entered as random effects in the Logistic Mixed Effect Model analysis on accuracy data. The effects of *item* and *participant* were not significant in the first analysis of the frequency effect as well in the second analysis of the lexicality effect (all Zs < 1).

#### 3.1 Frequency effect: RTs and z-transformed data analyses

Table 2 report results of Age comparison analysis. As shown in table the main effects of *frequency* and *length* were significant for both raw and z-transformed data. The effect of age was significant in the raw data, whereas the effect of *language* was not significant. The *length* by *age* and the *length* by *language* interactions were significant: the length effect was stronger in younger than in older children and in Italian than in English children. The *length* by *language*, the *frequency* by *language* and the *frequency* by *length* by *language* interactions were significant. When over-additivity was controlled, the English children showed a larger frequency effect than the Italian children (especially for the 5- and 6-letter words, at least p < .05). A similar length effect was evident for Italian and English children for low-frequency words; for high-frequency words, length influenced Italian children more than English children. The *frequency* by *length* by *age* interaction was significant only in the raw data, not when over-additivity was taken into account.

Table 4 report results of *School-year comparison* analysis. The ANOVA showed significant main effects of *frequency* and *length*. The main effect of language was not significant. The *frequency* by *length* and the *frequency* by *length* by *language* interactions were significant only in the z-transformed data analysis. The frequency effect was larger in English children, particularly for 5- and 6-letter words. The length effect was significant for both high and low frequency words in the Italian children, but only for low frequency words in the English children (except for the 7-9-letter high-frequency words, which were read more slowly than shorter words).

#### 3.2 Frequency effect: Error analyses

Regarding Age *comparison* analysis, as shown in Table 3, the effects of *age*, *frequency*, *length* and *language* were significant. The main effect of language indicated higher percentage of errors for English than for Italian children (8.7% vs 7.1%, respectively). Several interactions were significant. The *age* by *frequency* by *language* interaction indicated smaller frequency effects with age for both languages, but particularly in the Italian children. The effect of age was reduced in the case of high frequency words, especially in English children (no difference between younger and older English children). The *length* by *frequency* by *language* interaction highlighted that, in Italian children, the number of errors increased as a function of length for both high and low frequency words, even though the effect was stronger for low frequency words. In English children, the frequency effect was large, but did not interact with length. The *length* by *age* by *language* interaction showed that length modulated the performance of both young and older Italian children, whereas length did not consistently affect the performance of older English children. The effects of items and participants were not significant (all zs < 1).

Regarding *School-year comparison*, Table 5 reports the significance of the effects of *frequency* and *length* but not *language*. Error rates were higher on low frequency words compared to high frequency words and with 7–9 letter words compared to 4-letter words. Also in this case, the random effects of items and participants were not significant (all zs < 1).

#### 3.3 Lexicality effect: RTs and z-transformed data analysis

Regarding *Age comparison* analysis, the ANOVAs on raw and z-transformed data showed main effects of *lexicality* and *length* (Table 2) as well as age (only in the raw data). The effect of language was significant in the raw data, indicating faster RTs for English than for Italian children (1209 vs 1438 ms). Several interactions were significant (particularly in the z-transformed data analysis) including the *lexicality* by *length* by *age* by *language* interaction. In the Italian children, length influenced both words and non-words, but the effect was much greater with the latter stimuli. Although performance improved with age, this general pattern remained unchanged. In the English sample, both lexicality and length influenced younger children; in the older children, there was still an effect of lexicality, but only a small effect of length.

In *Year-school comparison* ANOVA, the main effects of *lexicality* and *length* were significant, while the effect of *language* was not significant (see Table 5). Several interactions were significant, including the *lexicality* by *length* by *language* interaction. The Italian children showed a length effect for words (mean increase per letter = 52 ms) and non-words (193 ms). The English children showed smaller length effects for both words (36 ms) and non-words (38 ms). The lexicality effect was evident in both groups for each length examined (at least p < .0001). However, in the Italian children, it increased with stimulus length (from 187 to 611 ms passing from 4- to 7-9-letter stimuli); in the English children remained stable across lengths (mean lexicality effect = 420 ms).

#### 3.4 Lexicality effect: Error analyses

Table 3 report results of Age *comparison* analysis. ANOVA showed the significance of the effects of *age*, *lexicality*, *length* and *language* (11.2% vs 12.8% for Italian and English children, respectively). The third-order *lexicality* by *length* by *language* by *age* interaction was significant. The lexicality effect was larger in younger than older children across languages. In the younger samples, English children performed worse than Italian children with both types of stimuli, but especially with non-words. There were no overall language differences in the older children. The length effect was small and in the expected direction in Italian children, but absent in the English children. The random effects of items and participants were not significant (all zs < 1).

Regarding *School-year comparison* (see Table 5), the effects of *lexicality* and *length* were significant, but *language* was not. The *language* by *length* interaction was significant: while for Italian children the percentage of errors increase in function of word length, English children show this trend except for 6 letter words that were read more accurately than other words (see paragraph General data). Also in this case, all random effects were insignificant (all zs < 1).

#### 3.5 Summary of results

To provide an overview of the findings, Table 6 summarizes the length, frequency and lexicality effects in each sample of readers (for details on the computation of the effects see the legend of the table). The table highlights some major findings:

- In terms of accuracy, the frequency effect was larger in the young English than in the young Italian readers;
- The sizes of the frequency and lexicality effects were similar in the two language samples in older children, both in the chronological and school comparisons;
- As for reading speed, the young English and Italian children showed similar length effects; in older children, the length effect was larger in Italian than in English children (length effects were not always detectable in English children in terms of accuracy);
- In older, English children, length did not interact with lexicality and frequency as it did in the Italian sample; *i*.*e*., Italian children showed smaller length effects for high frequency words compared to non-words and low frequency words (a pattern present for both accuracy and speed irrespective of age).

**Table 6:** Summary of major findings. The mean length, frequency and lexicality effects are presented for each sample of readers in terms of accuracy (reading errors) and speed (in terms of z scores).

	YOUNGER CHILDREN		OLDER CHILDREN		
	Italian	English	Italian	Italian	English
Mean years of age	7–8	7–8	9–10	10–11	9–10
Grade	2nd	3rd	4th	5^th^	5th
	% Errors				
Frequency effect	6.9	11.7	2.9	2.7	1.9
Lexicality effect	17.2	20.2	8.8	10.3	9.6
Length effect					
HF words	0.0	4.7	2.0	0.6	1.4
LF words	4.1	3.5	0.5	1.9	-1.1
Non-words	0.9	6.2	1.5	2.1	1.5
Notes	Frequency and lexicality interacted with length	Length did not interact with frequency/ lexicality	Frequency and lexicality interacted with length		Length did not interact with frequency/ lexicality
	RT—Z scores				
Frequency effect	0.41	0.54	0.53	0.46	0.39
Lexicality effect	1.22	1.48	1.67	1.70	1.56
Length effect					
HF words	0.27	0.26	0.19	0.18	0.16
LF words	0.40	0.36	0.36	0.30	0.16
Non-words	0.54	0.54	0.53	0.82	0.06
Notes	Frequency and lexicality interacted with length				Length did not interact with frequency/ lexicality

Notes: Hf = High frequency; Lf = Low frequency. We computed the effects based on both speed and accuracy data; in the former case, we used z scores from condition means because they allow estimating the effects independent of the large increase in reading speed observed with age/reading experience. The frequency effect was computed by subtracting z scores/errors of low frequency words from those of high frequency words (with lengths collapsed): positive values indicated better performance on HF words. The lexicality effect was obtained by subtracting z scores/errors of non-words from those of (high frequency) words (with lengths collapsed). The length effect is the mean increase per letter in the case of non-words, low frequency and high frequency words, respectively.

## Discussion

Overall, the present results underscore the importance of examining not only errors, but also RTs when comparing reading acquisition across orthographies. Also, they indicate that reading acquisition is expressed by changes in general orthographic skills as well as differential sensitivity to specific psycholinguistic parameters as a function of age and orthographic consistency. Therefore, full consideration of all these factors appears critical to clarify differences and similarities in reading acquisition across languages such as English and Italian. We will discuss in turn differences in reading accuracy and speed, the role of a global component, and the specific effects of psycholinguistic parametears as a function of orthographic consistency. Then, we will attempt to integrate these findings to provide a comprehensive description of the reading profiles in the two languages.

### 1 Cross-linguistic differences in reading accuracy and speed

There is strong evidence that English children have disproportionate difficulty in the early stages of literacy compared with readers of regular orthographies [2]. In the present study, after five years of school, the early difficulties of English children seem partially resolved and differences in reading acquisition between the two languages are more qualitative than quantitative, with specific cross-linguistic differences as a function of the characteristics of stimuli and age.

In a parallel study [34], the present English and Italian samples were also examined in spelling (using the same stimuli). Results for spelling revealed much larger language effects than those reported here for reading. Across ages and conditions English children were always less accurate than Italian children. These results are generally in keeping with the idea that, in English, spelling is a more challenging than reading [35]. Together, the two studies indicate that the effect of orthographic inconsistency of English on literacy acquisition is more long lasting in spelling than in reading.

Across conditions, the younger Italian children read more slowly than the chronologically age-matched English children. Some studies reported English children to be faster [15,16,18] while others to be slower [13] than children reading regular orthographies. These inconsistencies may be partially due to the large inter-individual differences that characterize the English children and that may result in different average performances from one study to another. Increased variability was found in previous studies both when the English readers were faster [16,18] or slower [13] than readers of regular orthographies. A greater variability in English than Italian readers was also found among adults in a study using both the Rapid Serial Visual Presentation (RSVP) and vocal RT paradigm [36]. In the present study, we explored variability starting from the influences of global information processing effects. Contrary to the prediction of a global factor (i.e., larger RT variability in slower individuals [24,25]), our English sample was generally faster, but more variable than the Italian sample, suggesting that increased variability is a specific characteristic of English children and not a simple by-product of speed of orthographic decoding. This may, in fact, explain the reluctance of some researchers to use RTs with young English-speaking children (at conferences on reading, we often hear the comment: “*Reaction times don’t work well with English children*!”) and their preference for focusing only on accuracy. In small experimental samples, the proportion of English children who read slowly may be unstable and yield unreliable results. With this caveat, however, some characteristics of reading performance emerge clearly only when one considers RTs.

In discussing general performance differences, it should be kept in mind that English and Italian orthographies have quite different structural characteristics. The two languages not only differ for grapheme-to-phoneme consistency, but also for complexity of syllabic structure. In English, only 5% of monosyllables are consonant-vowel CV [37], while in Italian CV is the most frequent syllable type, covering 56% of syllable tokens in written corpora [38]. The lower syllabic complexity of Italian makes it easier to segment words into phonemes/syllables and, in turn, to acquire grapheme-to-phoneme mappings. Moreover, the embedding of grapheme-phoneme correspondences in consonant clusters might make it difficult to acquire these correspondences. For example, Seymour *et al*. [2] found that syllabic complexity affects accuracy and speed of reading non-words (but not familiar words) and exaggerates the lexicality effect. On the other hand, word length and number of syllables are higher in Italian (*e*.*g*., the mode length in the Italian lexicon is 4 syllables [39]) than English, and this increases the demands for visual analysis and phonological buffering [40]. The greater number of syllables in the Italian language might contribute to the greater slowness of Italian children compared to English speaking children. However, evidence for a contribution of syllable number to reading aloud is limited to lower frequency words and nonwords [41,42], and to our knowledge evidence for an effect of the number of syllables on children’s reading aloud is lacking. It is important to remind that we used lists of words that were comparable for several characteristics, although not for number of syllables or syllabic complexity. To allow comparison, our distributions of stimuli were not typical of the two languages. English children were presented with stimuli which were only regular and longer words were over-represented. In contrast, Italian children were presented with stimuli which did not include any morphologically complex words which are very common among Italian long words.

A further note of caution concerns cross-linguistic differences in teaching programs and in the characteristics of school systems. Although in recent years a phonic approach has been increasingly used in English schools, teaching strategies might still be different in the two languages, with a stronger emphasis on acquiring sight vocabulary in English. Moreover, English children begin formal instruction one year before Italian children. We tried to control for these discrepancies by matching for both age and level of schooling, but we do not know to what extent earlier teaching might contribute to the differences observed.

Finally, it is important to point out that the Italian and English samples had different sizes and the smaller number of English participants might have influenced the obtained findings. Future studies seem necessary to replicate the present cross-linguistic differences.

### 2 Role of global components in reading acquisition in the two languages

With experience, children’s reading proficiency improves with all visually presented linguistic materials. For example, consider the superior performance of older children of both languages in reading non-words, *i*.*e*., stimuli they were not exposed to during their school years. Thus, improvement with age/experience in any given condition can be viewed as a joint effect of global and specific components. We tackled this question by referring to the RAM [24] and the DEM [25]. Together these models allow identifying global components in the data and examining the influence of specific effects controlling for the effect of global components.

Our results indicate that a global factor influences the reading acquisition of both English and Italian children. As predicted, in both languages reading speed improved with age proportionally across conditions and standard deviations co-varied linearly with performance in the respective conditions. These data are consistent with previous findings on Italian children [23,43] and extend them to English children. Nevertheless, the global factor accounted for different proportions of variance in the two languages. In Italian children, a proportionally similar increase in reading performance was found for all types of stimuli, indicating a large role of the global factor. By contrast, in English children the increase in performance with age was not entirely explained by the global factor, indicating the greater influence of additional specific variables.

Studies of Italian dyslexic and proficient readers tried to characterize the nature of the global factor by identifying the conditions (in terms of both task and type of stimuli) that fit the predictions of a global factor versus those that do not. In dyslexic readers, tasks that require the processing of a letter string (*i*.*e*., reading and lexical decision) load on the global factor regardless of whether they involve words or pronounceable and unpronounceable non-words [44, 45, 46]. Similar results were obtained in a study examining reading development in proficient readers [23]. By contrast, tasks requiring the identification or matching of single graphemes or bigrams [46, 47] and tasks requiring the identification of pictures [45] did not consistently load on this global factor. Finally, the global factor accounted for performance on words and non-words presented in the visual modality, but not for processing the same stimuli in the auditory modality [43]. These results indicate a key role of the *ability to visually process a string of letters with or without lexical value* in explaining reading variability. We proposed that this global factor identifies a pre-lexical orthographic analysis of the visually presented letter string and we linked this factor to *speed of orthographic decoding*.

A neural model that accounts for the encoding of the graphemic string has been proposed by Dehaene et al. [48]. The Local Combination Detector model posits that written words are encoded by a hierarchy of detectors tuned to increasingly larger and more complex word fragments (visual features, single letters, bigrams, quadrigrams and, possibly, words). At the neural level, information from letter features and single letters converges on the so-called visual word form area (VWFA). Importantly, several studies found that dyslexic individuals show selective hypo-activation of the VWFA (for a review see [49]).

If this interpretation is true, the present data indicate that the contribution of speed of orthographic decoding is different in the two languages. The Italian language has many long, multi-syllabic, morphologically complex words. Reading these words implies analyzing a complex visual stimulus before decoding can take place. By contrast, the English language has a much larger incidence of short words and irregular words. A component tapping orthographic analysis may account for less variation, because developing an orthographic representation of short words is easier and because reading irregular words relies on a different factor (lexical knowledge) for correct processing. As discussed below, in English reading proficiency requires larger processing units than in Italian. Being able to shift to some ‘parallel’ processing strategy will be an additional factor accounting for important inter-individual variability in the English data, but it would be less important in Italian (see below).

### 3 Reading strategies in the two languages: Evidence from psycholinguistic effects

By taking into account the influence of global components we were able to reliably evaluate the specific roles of psycholinguistic parameters over and above the effect of over-additivity. Results indicate similarities and differences in the reading profiles of English and Italian children as a function of reading experience. From the start, English and Italian children showed different patterns, with the English children showing stronger effects of frequency (particularly in terms of accuracy), indicating greater reliance on whole-word processing. Because of the many orthographic inconsistencies in English, from the time English-speaking children start reading they need to use specific word representations and are induced to rely on orthographic lexical representations earlier than Italian children. Effects of length were more similar across languages in the younger children. However, in the older English children length effects disappeared almost completely, while they were preserved in the Italian children. This indicates that a parallel processing strategy is more extensively adopted by English readers at the end of primary school. This result fits well with the existing literature. Length effects have not been reported systematically among English-speaking adults (e.g., [50]) except in the case of powerful experimental designs using a very large number of items and a large range of lengths [51,52]. By contrast, in regular orthographies (and particularly in Italian) length effects are observed in both children [53] and adults [54,55].

The psycholinguistic grain size theory [1] proposes that cross-linguistic variations reflect differences in the units used for phonological recoding. Children discover the most efficient grain size in a given orthography to improve their reading. In regular orthographies, reading output is primarily based on grapheme-phoneme correspondence because mapping at this level is simple and direct. By contrast, in irregular orthographies the use of these small units may result in errors; thus, children use larger (and less inconsistent) chunks, such as patterns of letters, rhymes, syllables, or even whole words [12,56]. The need to develop lexical representations earlier fits well with a processing strategy favouring larger orthographic units and a parallel processing mode. Present findings are also consistent with a recent cross-linguistic study that compared eye movements of English and German children in reading target words included in sentences [57]. Greater reliance on small-unit decoding was detected as a function of orthographic consistency: children’s gaze durations indicated stronger effects of word length in German compared to English children during first-pass reading. By contrast, English children generally used larger grain size units during first-pass reading but they relied on small-unit decoding only upon rereading. When the subsequent sentence context did not confirm their first reading attempt, then the eyes moved back to the target word and more analytic decoding was used.

Despite Italian children prevalently used small grain sizes of analysis, they did not rely exclusively on grapheme-phoneme conversion in reading [58]: larger units of analysis were used for known words in order to improve reading speed. In fact, Italian children showed clear frequency and lexicality effects, which indicate the use of larger grain sizes, as shown in the present research and in several previous studies on Italian children [59,60,61] and adults (e.g., [62]). Moreover, there is evidence that, when children do not possess the whole-word representation of a given word, as it may be the case for low frequency words, units of analysis smaller than the whole word but larger than the single phoneme can be used. For example, various studies demonstrated that morpheme-based reading is available and efficient in Italian developing readers, and facilitates reading already by 2^nd^ grade (for a review see [63]). Thus, it seems that Italian children also use reading units of a large grain sizes although to a lesser degree than English speaking children.

Reliance on larger processing units and a more parallel processing mode well accounts for the fact that English children are faster as well as more variable. In the English children, variability might be related to two factors: (a) speed of orthographic decoding, which improves more or less uniformly with age across individuals and conditions and (b) the ability to use a parallel mode of processing. The availability of lexical representations may be a second source of improvement in the English sample which increases the overall variability. Consistent with this hypothesis, the slowest quartile of English children showed marked effects of frequency and lexicality, indicating large differences in the reliance on reading units of large grain sizes. This second source of variability might be less important in the Italian children because they rely on a more analytic processing mode. Therefore, most of the inter-individual variability in the Italian children depends on differences in orthographic processing, as indexed by the global factor, with smaller contributions made by differences in lexical expansion. Hanley *et al*. [15] also examined the reading performance of the four quartiles in English and Welsh children and found larger cross-linguistic differences between the quartiles with poorer reading skills (see also [14] for similar results). The present findings confirm a difference in the distribution of reading performances as a function of orthographic consistency. The poorest-performing quartile appreciably differed from the others only in English (but not in Italian) children. Thus, it could be supposed that, due to the characteristics of the orthography, the presence of reading disorders might be less evident in Italian- than in English-speaking children.

Note that in this study we did not examine the role of semantic variables in modulating reading in the two languages. According to the Triangle Model ([64]; see also [65] for evidence in neuroimaging studies) an indirect semantically mediated pathway (in which the translation from orthography to phonology is mediated through access to semantic knowledge) provides additional support for reading, which is especially important for exception words (that are poorly served by the orthography-to-phonology pathway). Then, it is possible to presume a larger reliance on the semantically mediated pathway in English-speaking children compared to Italian readers. Further research is needed to evaluate this possibility.

Our study highlights qualitative differences in the acquisition of reading performance as a function of orthographic consistency. Whether or not different models of reading are developed for regular and irregular orthographies, examination of reading profiles across languages may be a powerful tool for reading research.

## Appendix

Italian and English lists of high frequency words, low frequency words and non-words (for each length subset N = 10). Note that non-words were derived from high frequency words (non-words are reported in the same order as their paired high frequency words). For each word set, the average number of phonemes and syllables is reported.

**ITALIAN HIGH FREQUENCY WORDS:**
**4- letters** (phonemes = 4, sd = 0; syllables = 2.0, sd = 0): Zona, Mago, Erba, Seta, Lato, Base, Foto, Male, Gara, Arte;
**5- letters** (phonemes = 4.8, sd = 0.4; syllables = 2.0, sd = 0): Vasca, Barba, Sedia, Ansia, Bomba, Latte, Sonno, Ombra, Festa, Fuoco;
**6- letters** (phonemes = 5.6, sd = 0.5; syllables = 2.6, sd = 0.5): Matita, Sabbia, Nipote, Moneta, Affare, Fretta, Denaro, Sangue, Estate, Gruppo;
**7-9- letters** (phonemes = 6.8, sd = 1.0; syllables = 3.0, sd = 0.5): Femmina, Negozio, Inverno, Soffitto, Ghiaccio, Finestra, Silenzio, Ospedale, Famiglia, Bicchiere.

**ITALIAN LOW FREQUENCY WORDS:**
**4- letters** (phonemes = 4, sd = 0; syllables = 2.0, sd = 0): Rapa, Bava, Sega, Elmo, Orma, Lode, Zelo, Boia, Lino, Nuca;
**5- letters** (phonemes = 4.4, sd = 0.7; syllables = 2.0, sd = 0.0): Gobba, Manzo, Garza, Muffa, Sorso, Rospo, Sacca, Astro, Rissa, Aglio;
**6- letters** (phonemes = 5.1, sd = 0.7; syllables = 2.4, sd = 0.5): Fibbia, Dogana, Seppia, Biscia, Ovatta, Laccio, Cipria, Mancia, Avorio, Flotta;
**7-9- letters** (phonemes = 7.5, sd = 0.8; syllables = 3.3, sd = 0.5): Lampone, Bacheca, Diluvio, Cipresso, Aragosta, Lanterna, Merluzzo, Cisterna, Arsenale, Monastero.

**ITALIAN NON-WORDS:**
**4- letters:** Zila, Muci, Esfi, Siba, Libo, Bipo, Fipo, Mafe, Gomi, Urse (phonemes = 4.0, sd = 0; syllables = 2.0, sd = 0);
**5- letters:** Nisca, Bilfa, Sipio, Arsia, Bumbo, Libbe, Sinno, Ostra, Fisti, Fuago (phonemes = 4.9, sd = 0.6; syllables = 2.0, sd = 0);
**6- letters:** Matoba, Sibbio, Nulote, Minefa, Affime, Fietta, Deparo, Sancio, Espele, Chippo (phonemes = 5.4, sd = 0.7; syllables = 2.6, sd = 0.5);
**7-9- letters:** Reffina, Nagopio, Isterno, Soffatto, Ghioggia, Fibestre, Sipinzio, Ostedane, Firiglia, Bucchiole (phonemes = 6.8, sd = 1.0; syllables = 3.0, sd = 0.5).

**ENGLISH HIGH FREQUENCY WORDS:**
**4- letters** (phonemes = 3.2, sd = 0.6; syllables = 1.0, sd = 0.3): Card, Club, Farm, Game, Lady, Milk, Neck, Roof, Sand, Soil;
**5- letters** (phonemes = 4.1, sd = 0.3; syllables = 1.5, sd = 0.7): Clock, Cloth, Flame, Paper, Party, Plane, Pound, Radio, Storm, Table;
**6- letters** (phonemes = 4.9, sd = 0.7; syllables = 2.0, sd = 0.5): Border, Branch, Family, Jungle, Letter, Number, Office, Silver, Volume, Winter;
**7-9- letters** (phonemes = 6.5, sd = 1.1; syllables = 2.5, sd = 0.5): Article, Blanket, Example, Husband, Morning, Opinion, Partner, Distance, Hospital, Direction.

**ENGLISH LOW FREQUENCY WORDS:**
**4- letters** (phonemes = 3.7, sd = 0.5; syllables = 1.0, sd = 0.0): Ramp, Mink, Plum, Scar, Cork, Brim, Cube, Crib, Wink, Sage;
**5- letters** (phonemes = 3.9, sd = 0.6; syllables = 1.4, sd = 0.5): Clown, Moose, Latch, Pedal, Spice, Trash, Spade, Cider, Gravy, Maple;
**6- letters** (phonemes = 4.8, sd = 0.8; syllables = 1.9, sd = 0.3): Goblet, Cavern, Bandit, Buckle, Pimple, Stripe, Pickle, Hurdle, Beetle, Napkin;
**7-9- letters** (phonemes = 6.9, sd = 1.3; syllables = 2.7, sd = 0.7): Emerald, Hamster, Lobster, Inferno, Scooter, Scorpion, Lemonede, Pendulum, Ornament, Dandelion.

**ENGLISH NON-WORDS:**
**4- letters:** Sarn, Klib, Nard, Zale, Maly, Gilm, Leck, Zoof, Dant, is (phonemes = 3.5, sd = 0.5; syllables = 1.2, sd = 0.4);
**5- letters:** Glock, Ploth, Glame, Naver,Narvy, Flape, Cound, Panio, Stoln, Saple (phonemes = 4.2, sd = 0.4; syllables = 1.4, sd = 0.7);
**6- letters:** Vorner, Tranch, Daminy, Jumple, Retter, Zunder, Ollice, Dilber, Colune, Sinter (phonemes = 5.0, sd = 0.7; syllables = 2.0, sd = 0.5);
**7-9- letters:** Andicle, Clandet, Exandle, Huscant, Horping, Omidion, Dartyer, Bisbance, Gosbital, Tinection (phonemes = 6.6, sd = 1.0; syllables = 2.6, sd = 0.5).

## Supporting Information

S1 Table Slope and r2 for each experimental condition. The mean RTs of each child in each condition have been plotted against the standard deviation for the same condition. The Appendix reports the slopes of these relationships as well as the r2 for each sample. Note the presence of very similar relationships across ages and languages. For each condition, and across languages, the individual standard deviation was predicted by the mean RTs. The faster children were also the least variable, as expected.
(DOCX)
