# Supplementary material for: Costs and Benefits of Orthographic Inconsistency in Reading: Evidence from a Cross-Linguistic Comparison
Source: PLoS One. 2016 Jun 29;11(6):e0157457. doi: 10.1371/journal.pone.0157457 (PMC4927093; doi:10.1371/journal.pone.0157457)
Supplement: S1 Table — The mean RTs of each child in each condition have been plotted against the standard deviation for the same condition. The Appendix reports the slopes of these relationships as well as the r2 for each sample. Note the presence of very similar relationships across ages and languages. For each condition, and across languages, the individual standard deviation was predicted by the mean RTs. The faster children were also the least variable, as expected. (DOCX) [file pone.0157457.s001.docx]

|  | | Italian children | | | | | | English children | | | |
| --- | --- | --- | --- | --- | --- | --- | --- | --- | --- | --- | --- |
|  |  | Younger children  (2nd grade) | | Older children  (4th grade) | | Year school comparison (5th grade) | | Young children  (3rd grade) | | Older children  (5th grade) | |
|  |  | B | r^2^ | B | R^2^ | B | r^2^ | B | r^2^ | B | r^2^ |
| Non-words | 4 letters | 0.43 | 0.73 | 0.58 | 0.65 | 0.56 | 0.89 | 0.38 | 0.55 | 0.40 | 0.69 |
|  | 5 letters | 0.39 | 0.70 | 0.50 | 0.65 | 0.51 | 0.80 | 0.46 | 0.59 | 0.46 | 0.75 |
|  | 6 letters | 0.38 | 0.77 | 0.42 | 0.71 | 0.60 | 0.78 | 0.38 | 0.64 | 0.37 | 0.80 |
|  | 7-9 letters | 0.40 | 0.68 | 0.35 | 0.70 | 0.39 | 0.26 | 0.28 | 0.52 | 0.28 | 0.51 |
| Low frequency words | 4 letters | 0.65 | 0.76 | 0.67 | 0.75 | 0.64 | 0.84 | 0.68 | 0.89 | 0.66 | 0.75 |
|  | 5 letters | 0.56 | 0.68 | 0.61 | 0.71 | 0.54 | 0.74 | 0.61 | 0.93 | 0.69 | 0.93 |
|  | 6 letters | 0.56 | 0.68 | 0.61 | 0.71 | 0.54 | 0.74 | 0.61 | 0.93 | 0.69 | 0.93 |
|  | 7-9 letters | 0.84 | 0.63 | 0.52 | 0.53 | 0.54 | 0.67 | 0.36 | 0.70 | 1.06 | 0.89 |
| High-frequency words | 4 letters | 0.68 | 0.63 | 0.64 | 0.67 | 0.52 | 0.62 | 0.64 | 0.63 | 0.89 | 0.93 |
|  | 5 letters | 0.54 | 0.68 | 0.68 | 0.86 | 0.55 | 0.70 | 0.89 | 0.91 | 0.80 | 0.98 |
|  | 6 letters | 0.48 | 0.65 | 0.51 | 0.76 | 0.56 | 0.78 | 0.87 | 0.96 | 0.58 | 0.82 |
|  | 7-9 letters | 0.78 | 0.78 | 0.40 | 0.46 | 0.46 | 0.83 | 0.61 | 0.84 | 0.82 | 0.80 |
|  | | | | | | | | | | | |
